# Hijacking translation in addiction

**DOI:** 10.7554/eLife.14576

**Published:** 2016-03-01

**Authors:** Alicia Izquierdo, Alcino J Silva

**Affiliations:** 1 Department of Psychology, the Brain Research Institute, the Integrative Center for Learning and Memory, and the Integrative Center for Addictions, University of California Los Angeles, Los Angeles, United States; 2 Departments of Psychology, Neurobiology and Psychiatry, the Brain Research Institute, and the Integrative Center for Learning and Memory, University of California Los Angeles, Los Angeles, United Statesalcinojsilva@gmail.com

**Keywords:** nicotine-induced plasticity, ventral tegmental area, protein synthesis, cocaine, Human, Mouse

## Abstract

Two studies suggest that the reduced activity of a translation initiation factor called eIF2α might be partly responsible for the increased risk of drug addiction seen in adolescents.

**Related research articles** Placzek AN, Molfese DL, Khatiwada S, Viana Di Prisco G, Wei H, Sidrauski C, Krnjević K, Amos CL, Ray R, Dani JA, Walter P, Salas R, Costa-Mattioli M. 2016. Translational control of nicotine-evoked synaptic potentiation in mice and neuronal responses in human smokers by eIF2α. *eLife* **5**:e12056. doi: 10.7554/eLife.12056

Huang W, Placzek A, Viana Di Prisco G, Khatiwada S, Sidrauski C, Krnjević K, Walter P, Dani JA, Costa-Mattioli M. 2016. Translational control by eIF2α phosphorylation regulates vulnerability to the synaptic and behavioral effects of cocaine. *eLife* **5**:e12052. doi: 10.7554/eLife.12052

**Image** The brains of smokers and non-smokers respond differently to rewards

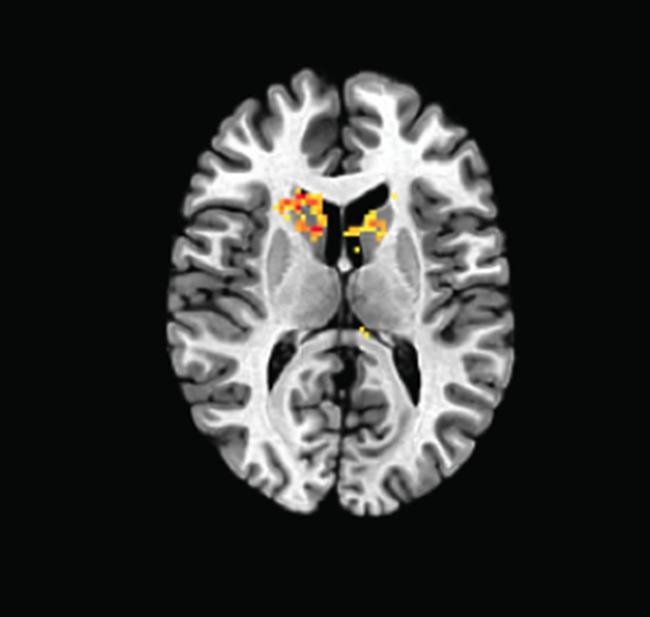

Exposure to drugs of abuse – such as nicotine and cocaine – changes the brain in ways that contribute to the downward spiral of addiction. Adolescents are especially vulnerable since their newly found independence is often associated with taking more risks (Spear, 2000). To make matters worse, adolescence is also characterized by an increased sensitivity to natural rewards and drugs of abuse (Badanich et al., 2006; Brenhouse and Andersen, 2008; Stolyarova and Izquierdo, 2015). Experiences with illicit substances alter the genes that are expressed in the brain, and lead to increased consumption of these substances. To date much of the work that has characterized this insidious cycle has focused on changes in gene activation, or modifications to proteins that have already been produced (Robison and Nestler, 2011). By comparison, much less is known about how changes in protein synthesis might contribute to addiction.

Exposure to cocaine leads to persistent changes in the part of the brain that releases the chemical dopamine. Specifically, alterations to a part of the midbrain called the ventral tegmental area (VTA), along with its connections to other regions of the brain, are thought to mediate the transition from recreational to compulsive drug use and subsequently to addiction (Luscher and Malenka, 2011). Drugs of abuse make the neurons in the VTA more excitable overall. The drugs do this by altering two opposing processes – both of which involve the translation of messenger RNAs to produce new proteins – in ways that ultimately strengthen the connections between neurons (Ungless et al., 2001; Lüscher and Huber, 2010).

Now, in two papers in eLife, Mauro Costa-Mattioli from the Baylor College of Medicine and colleagues report that a protein that regulates translation is also responsible for much of the increased risk of addiction seen in adolescent mice and humans. The protein of interest is a translation initiation factor called eIF2α.

In the first paper, Wei Huang, Andon Placzek, Gonzalo Viana Di Prisco and Sanjeev Khatiwada – who are all joint first authors – and other colleagues report that adolescent mice are more vulnerable to the effects of cocaine compared to adult mice (Huang et al., 2016). They could measure this effect as changes in both the behavior of the mice and in the two opposing processes that affect the strength of the connections between neurons.

Cocaine greatly reduced the levels of the phosphorylated form of eIF2α in the VTA of adolescent mice, while adult mice were less affected. Phosphorylation of eIF2α changes its activity, and Huang et al. next explored if this difference might explain why adolescents are more sensitive to cocaine. In support of the idea, they found that adult mice could be made more sensitive to cocaine if the levels of phosphorylated eIF2α were reduced. Similarly, in other experiments, adolescent mice could be rendered more adult-like if their levels of phosphorylated eIF2α were increased.

Huang et al. also report that phosphorylated eIF2α promotes the synthesis of a protein called OPHN1; this protein is known to reduce the strengthening of neural connections that is also typically linked to an increased sensitivity to drugs of abuse. So, Huang et al. showed that decreases in phosphorylated eIF2α during adolescence lead to lower levels of OPHN1, which could explain adolescents’ increased risk of drug addiction.

Huang et al. also demonstrated that other abused drugs that act quite differently in the brain from cocaine (i.e. methamphetamine, nicotine and alcohol) also decrease the levels of phosphorylated eIF2α in the VTA of adult mice. Thus, they appear to have uncovered a general mechanism by which exposure to drugs affects protein synthesis, changes the connections between neurons, and leads to behaviors associated with addiction.

In the second paper, Placzek, Khatiwada, fellow co-first author David Molfese, and other colleagues probed nicotine’s effects on the phosphorylation levels of eIF2α (Placzek et al., 2016). Similar to the cocaine results, a low-dose of nicotine in adolescent mice triggered increased signs of addiction in the VTA. Furthermore, reducing the level of phosphorylated eIF2α in adult mice made the neurons in the VTA more sensitive to nicotine’s effects.

Placzek et al. then used functional magnetic resonance imaging with a group of human volunteers, and found a variation in the gene for eIF2α that was related to how much cigarette smokers in the group responded to a reward. The variant reduces the expression of the eIF2α protein, and this finding suggests that the same translation-based mechanism underlies addiction in different species (i.e. in both mice and humans). Further work is now needed to explain how these changes in the expression of eIF2α lead to the changes in brain activity seen in addiction. Since mice with reduced phosphorylated eIF2α levels are more susceptible to nicotine-induced changes in the brain that underlie addiction, individuals with the genetic variant may also be more likely to show addictive behaviors.

The two papers by Costa-Mattioli and colleagues demonstrate that eIF2α is a promising new target for the treatment of addiction. Its role in nicotine addiction is highly relevant given that e-cigarettes are a widely used tobacco product amongst adolescents (Miech et al., 2015). As with all important discoveries, these new findings raise a number of questions. For example, are the effects of eIF2α in addiction specific to the VTA, or are other regions of the brain involved (Jian et al., 2014)? Does eIF2α also affect other aspects of addiction such as relapse? Further work could probe if phosphorylated eIF2α regulates the synthesis of other proteins, beyond OPHN1, that may also have a role in the addiction process.

Finally, increased concentrations of phosphorylated eIF2α have been found in patients suffering from neurodegenerative diseases such as Alzheimer’s, Parkinson’s and Huntington’s disease (Ma et al., 2013; Moreno et al., 2012; Leitman et al., 2014). Is there evidence for changes in addiction behaviors in the very early stages of these diseases? Protein synthesis is important for memory, and the VTA also plays a central role in learning and memory. As such, could changes in phosphorylated eIF2α in the VTA affect memory processes? This might suggest that the hijacking of phosphorylated eIF2α by substances of abuse goes well beyond addiction and affects fundamental cognitive processes such as memory.
